# Inferring Atmospheric Particulate Matter Concentrations from Chinese Social Media Data

**DOI:** 10.1371/journal.pone.0161389

**Published:** 2016-09-20

**Authors:** Zhu Tao, Aynne Kokas, Rui Zhang, Daniel S. Cohan, Dan Wallach

**Affiliations:** 1 Department of Computer Science, Rice University, Houston, Texas, United States of America; 2 Department of Media Studies, University of Virginia, Charlottesville, Virginia, United States of America; 3 Department of Civil and Environmental Engineering, Rice University, Houston, Texas, United States of America; Universidade de Vigo, SPAIN

## Abstract

Although studies have increasingly linked air pollution to specific health outcomes, less well understood is how public perceptions of air quality respond to changing pollutant levels. The growing availability of air pollution measurements and the proliferation of social media provide an opportunity to gauge public discussion of air quality conditions. In this paper, we consider particulate matter (PM) measurements from four Chinese megacities (Beijing, Shanghai, Guangzhou, and Chengdu) together with 112 million posts on Weibo (a popular Chinese microblogging system) from corresponding days in 2011–2013 to identify terms whose frequency was most correlated with PM levels. These correlations are used to construct an Air Discussion Index (ADI) for estimating daily PM based on the content of Weibo posts. In Beijing, the Chinese city with the most PM as measured by U.S. Embassy monitor stations, we found a strong correlation (R = 0.88) between the ADI and measured PM. In other Chinese cities with lower pollution levels, the correlation was weaker. Nonetheless, our results show that social media may be a useful proxy measurement for pollution, particularly when traditional measurement stations are unavailable, censored or misreported.

## Introduction

User generated social media data are widely seen as an important source for the observations of crowds to be harnessed for a variety of applications. Perhaps most famously, Google’s “Flu Trends” project found a significant correlation between localized search engine queries related to illness symptoms and the subsequent growth in emergency room visits [1–2]. Internet data are used to understand public sentiment for a variety of applications, ranging from consumer marketing to stock trading [3–7]. Furthermore social media like Twitter, Facebook and Weibo provide a wide range of content and relatively precise timestamp, facilitating time series research for acute and chronic conditions [8–20].

One such condition that could be characterized by social media data is air quality. Ambient air pollution is estimated to kill 3.7 million people per year worldwide [21]. Most of these deaths occur due to fine particulate matter (i.e., PM_2.5_, denoting particles with aerodynamic diameter less than 2.5 microns), for which concentrations are especially high in cities of China. Ground-level measurements provide limited information about air quality in China, due to sparsity of monitors and withholding of some data [22–24]. Although capabilities are improving for measuring air quality from space, satellites face limitations for characterizing short-term variability in conditions at the surface [25]. Meanwhile, people frequently comment about the weather, a propensity that extends beyond spoken conversations to the online world. Air quality is thus an area that is well suited for investigating the potential for social media-based indicators to infer ambient conditions.

Here, we show how messages publicly posted to Weibo, the most popular microblogging service in China with over 300 million users [26], can be used to construct an “Air Discussion Index” (ADI) characterizing air quality conditions. Our approach derives the terms whose use correlates most directly with pollution metrics, rather than a *priori* selection of terms. Building upon prior data mining of Weibo messages [27], we show how meaningful inferences about pollutant conditions can be extracted from noisy social network data, despite the difficulty of computer processing for Chinese text.

## Material and Methods

### Collection of Weibo Posts and Air Quality Data

A statistical technique is used to identify words or phrases most associated with varying air quality conditions. An index of those terms, the ADI, can then be used to characterize the relationship between PM_2.5_ and social media posts.

For Weibo, we utilize public timeline posts collected for July 23, 2011 to May 15, 2013 from our previous research [28–29]. The public timeline posts were queried roughly once every four seconds, for which the Weibo server returned roughly 200 recent posts responding to each request. Public timeline is one of the Application Programing Interface API (http://open.weibo.com/), a set of routines and standards for accessing Weibo database, provided by Weibo to access their posts. The posts returned by public timeline API can be considered as real-time random sampling from total posts population coming to Weibo system. In total 500 Gigabytes of collected data, including 112 million Weibo posts from the four cities with available PM_2.5_ measurements described below, were stored and processed on a four-node cluster using Hadoop [30] and HBase [31].

For PM_2.5_, we utilize air quality reports from monitoring stations located at U.S. embassies or consulates in four cities: Beijing, Shanghai, Guangzhou, and Chengdu. Air quality readings from the U.S. consulate in a fifth city, Shenyang, were excluded because their air quality data overlaps with our Weibo data by only one month. PM_2.5_ data are collected by crawling U.S. Embassy and consulate air quality Twitter accounts, which report PM_2.5_ and ozone readings and corresponding U.S. Environmental Protection Agency air quality index (AQI) values hourly and/or daily. Since the format and content of the posts changed several times over the time period of the sample, we compute average daily (12 a.m.–11 p.m.) PM_2.5_ from the hourly data. The number of days in each city with available PM_2.5_ data, which means we collected PM_2.5_ value from Twitter successfully, and successful collection of Weibo posts, along with the average number of collected posts on those days, is shown in Table 1.

**Table 1:** Information of Weibo posts and air quality condition in four mega cities of China for this study.

City	Twitter site (twitter.com)	Twitter reports starting date	Number of valid days	Average daily Weibo posts	Average daily PM_2.5_ concentration (μg/*m*^3^)
Beijing	../BeijingAir	Feb 19, 2009	528	89000	100.1
Shanghai	../CGShanghaiAir	May 15, 2012	281	83000	53.4
Guangzhou	../Guangzhou_Air	Jun 15, 2011	462	81000	54.5
Chengdu	../CGChengduAir	Jun 28, 2012	207	21000	93.4

Official Chinese measurement data of PM_2.5_ became available in 2013, when China began to regulate PM_2.5_ as a criteria pollutant with an ambient air quality standard. Given the short overlap period of those data and our Weibo posts, we use the Chinese PM_2.5_ data only for an inter-comparison to assess the representativeness of the U.S.-reported data.

### Key Term Extraction in Weibo Post

Even though our Weibo crawler returned only original posts and excludes retweets, roughly 10% of the captured posts were identical. The main sources of these identical posts are the “posting machines” which enable users to generate posts automatically. Two examples are PiPi Timing Machine (weibo.pp.cc/time/) and Weibo Tong (wbto.cn/). These identical posts create noise in terms of forming topical trends, which do not reflect users’ real opinion. To alleviate this noise, the MD5 message-digest algorithm [32] is used to remove identical posts.

Identifying words in Chinese is not an easy task for computer systems. Chinese is different from western languages in that there are no spaces between words in a sentence. Most commonly used Chinese words are composed of two or more characters. Analysis is complicated in social media by the frequent use of neologisms in online discourse [33]. The approach adopted here is to utilize n-grams [29], representing *n* consecutive Chinese characters in a sentence. Some of these sequences of characters form meaningful words, but most of them do not. Trigrams (*n* = 3) were used in a preliminary experiment on a subset of Beijing data, and achieved slightly better correlation than bigrams (*n* = 2). However, considering the vastly larger computational task to analyze trigrams (2 billion in our current Weibo database) instead of bigrams (40 million), we decided to report only the algorithm and performance of bigrams in this paper.

We examined each of the 40 million bigrams in our data database. Rather than using any subjective judgment to select the relevant bigrams, the selection is performed automatically by the algorithms described here. For each term, the number of posts containing is counted daily and aggregated separately by city according to the user’s registered city, denoted as *post_count (term*, *date*, *city)*. When a term shows up multiple times in a post, it is counted only once. The post count is then divided by the number of all posts in that city on that day in our dataset, *base_post_count (date*, *city)*, resulting in a fraction of the posts containing that term in a particular city and on a particular day:

$$f(term,\;date,\;city)=\frac{post\_count\;(term,date,city)}{base\_post\_count\;(date,city)}$$ (1)

For each city and each term, a linear regression model is built to infer daily PM_2.5_.

$${\left[P\right]}_{city}=\alpha_{0}+\alpha_{1}{\left[T\right]}_{city,term}+\varepsilon$$ (2)

where [*P*]_*city*_ is the vector of the daily PM_2.5_ concentration of a city, [*T*]_*city*,*term*_ is the term’s daily fraction vector in which each element is calculated in Eq 1, α_0_ and α_1_ are the parameters of the linear function, and *ε* is the error term.

Using the above linear regression with four-fold cross validation [34], we fit the model to four ¾ subsets in each city. Each per-term model is validated by measuring the correlation coefficient between the model’s estimates for the ¼ reserved points and U.S. Embassy reported PM_2.5_ for that city at those points. Thus we get four correlation coefficients for each term city pair, and the final score of each candidate term of a city is the mean of those four values. At last, the terms are sorted by this final score for each city to generate a Sorted Term List (STL).

### Computation of Air Quality Discussion Index (ADI)

A naive way aggregate the probability in each term set (TS) in the ADI for different day at different city is simply to sum them together as below:

$${ADI}_{date,city,TS}=\sum_{term\in TS}sign(term,city)\times f(term,date,city)$$ (3)

,where *sign*(*term*,*city*) is the sign of *α*_1_ in Eq 2, which is either +1 or -1, depending on whether the term is positively or negatively correlated with PM_2.5_.

However, since the term’s base frequencies may differ by orders of magnitude, the effect caused by terms with smaller frequencies will be overwhelmed by those with larger frequencies. Standardization is a method to eliminate the influence of high frequency term. Therefore, instead of using the probability directly, we calculate the standard score or the z-values [35] in each term’s daily probability vector (Eq 1) to the mean equal to 0 and the variance equal to 1, which is *norm*(*term*,*date*,*city*). Then we use this normalized vector to compute the ADI by summing the normalized term probability over a period of time and city:

$${ADI}_{date,city,TS}=\sum_{term\in TS}sign(term,city)\times norm(term,date,city)$$ (4)

Selected Weibo terms can be either positively (e.g. “haze”) or negatively (e.g. “blue sky”) correlate with air pollution condition.

### Determination of Term Set and Estimation of PM_2.5_

The algorithms for selecting the terms for the ADI term set (TS) and for evaluating the term set are introduced here as Algorithm 1 and Algorithm 2.

**Algorithm 1**: **Algorithm of ADI term set selection.**

ADI_TERM_SET_SELECTION(city):

  TS ← ∅; BestPerformance ← 0;

  For each term_i_ in top N of STL_city_

     Add term_i_ to TS;

     CurrentPerformance = Evaluate(TS, city)

     If CurrentPerformance > BestPerformance

             BestPerformance ← CurrentPerformance;

     else

             Remove term_i_ from TS;

Return TS;

From the previous step, we get a STL in which the terms are sorted by the mean correlations for each city. To decide how many terms from the STL should be included in the term set (TS), an incremental approach is applied (Algorithm 1). The first term in the STL is added to a candidate term set. The second term in the STL is added to the ADI set, only if this second term increases the ADI infer performance. In this manner, all terms in the STL are scanned in order, with an additional term added to the TS only if this term increases the TS infer performance measured by Algorithm 2.

**Algorithm 2**: **Algorithm of term set evaluation**

Evaluate(TS, city):

   Compute [ADI] from Eq 4

   Divide data points (both [P] (PM_2.5_ observations) and [ADI]) into four continues sections;

   [P_f_] and [ADI_f_] denote the portion of section f in [P] and [ADI] (f = {1, …, 4}).

   [*P*_*f*_] and [*ADI*_*f*_] denote the complementary vector of [P_f_] and [ADI_f_]

   For each section f

      Learn model parameters β_0_ *and* β_1_in Eq 5 with [*P*_*f*_] and [*ADI*_*f*_]

      [FitP_f_] ← Fit the learned model with data [ADI_f_]

      R_f_ ← Pearson correlation coefficient between [P_f_] and [FitP_f_];

   R = average of R_f_;

Return R;

By Algorithm 2, a TS is evaluated by using simple linear regression with four-fold cross validation. In four-fold cross-validation, the original dataset is partitioned into four equal sized sub-datasets. Three sub-datasets are used for training purposes, while the remaining single sub-dataset is kept as the validation data to test the model performance. Then, this four cross-validation process is performed four times, with each of the four sub-datasets used in turn as the validation data. Here, the average value of the four correlations between the model’s estimates and reported PM_2.5_ concentrations is assigned as the score for that specified TS. The TS with the highest score becomes the Final Term Set (FTS), which will be used to compute the ADI.

After we decide the term set to compute ADI, PM_2.5_ is inferred from it by applying a linear model analogous to Eq 2, as shown in Eq 5.

$${\left[P\right]}_{city}=\beta_{0}+\beta_{1}{\left[ADI\right]}_{city,TS}+\varepsilon$$ (5)

Here [*P*]_*city*_ is the vector of daily PM_2.5_ as in Eq 2; [*ADI*]_*city*,*TS*_ is a daily aggregated normalized term vector whose elements are calculated as shown in Eq 4; *β*_0_ and *β*_*1*_ are the parameters of the linear function; and *ε* is the error term.

### Ethics Statement

The protocol of data processing and anonymization was followed by the standards of ethical conduct in Rice University. All Weibo posts used for analysis in this study were from our previous dataset [28], which crawled social media information available to the broad public. No attempt was made to inform Weibo users of the current study. In compliance with the privacy/ethic requirements of Sina Weibo Term of Service, the individual profiles were treated as encrypted sensitivity information and only the aggregate statistic results are reported. The raw data were de-identified before the current analysis began.

## Results and Discussion

### Weibo Terms Associated with PM_2.5_

The simple linear regression described in the methodology section is applied to the four cities in Table 1. The last five months of data (January 1, 2013—May 15, 2013) are left out as a validation data set for the overall performance evaluation. A total of 513,537 bigrams were identified for which the frequency of appearance in the total dataset was above a once-per-day threshold. A list with the highest scoring terms, sorted by the mean correlations, was produced for each city (see S1 File for the final term set for each city).

Many of the terms positively correlated with PM_2.5_ appear related to high air pollution levels, including dust, cough and mask. Many of the negatively correlated terms relate to good air quality; examples include the terms sunshine, rain, and blue. By looking at the sign of *α*_1_ in Eq 2, terms are classified into two categories objectively.

The most strongly positively correlated term for Beijing is “雾蒙” (misty foggy) while for Guangzhou it is “灰霾” (dust-haze). The bigram “不健” (part of “不健康”, unhealthy) topped the term set for Shanghai. Chengdu’s most correlated term “的天” (-like day), has a less obvious connection to air pollution, but it is a common connection phrase in Chinese to most likely describe the condition of air turbidity such as blue sky (negative correlate) or foggy sky/muddy sky (positive correlate). For non-Chinese speaking readers, we provide some descriptions of those terms. The bigrams are not necessarily words, for example “rain”, which have some specific meanings. Instead, bigrams are pairs of Chinese characters which appeared next to another in Weibo corpus, for example “s raini”. As mentioned earlier, there are no spaces in between the words like English, so in Chinese “s raini” would further become “sraini”. Since these bigrams cannot be associated directly with dictionary words, human interpretation is needed to examine the meaning of those bigrams.

**Fig 1:**
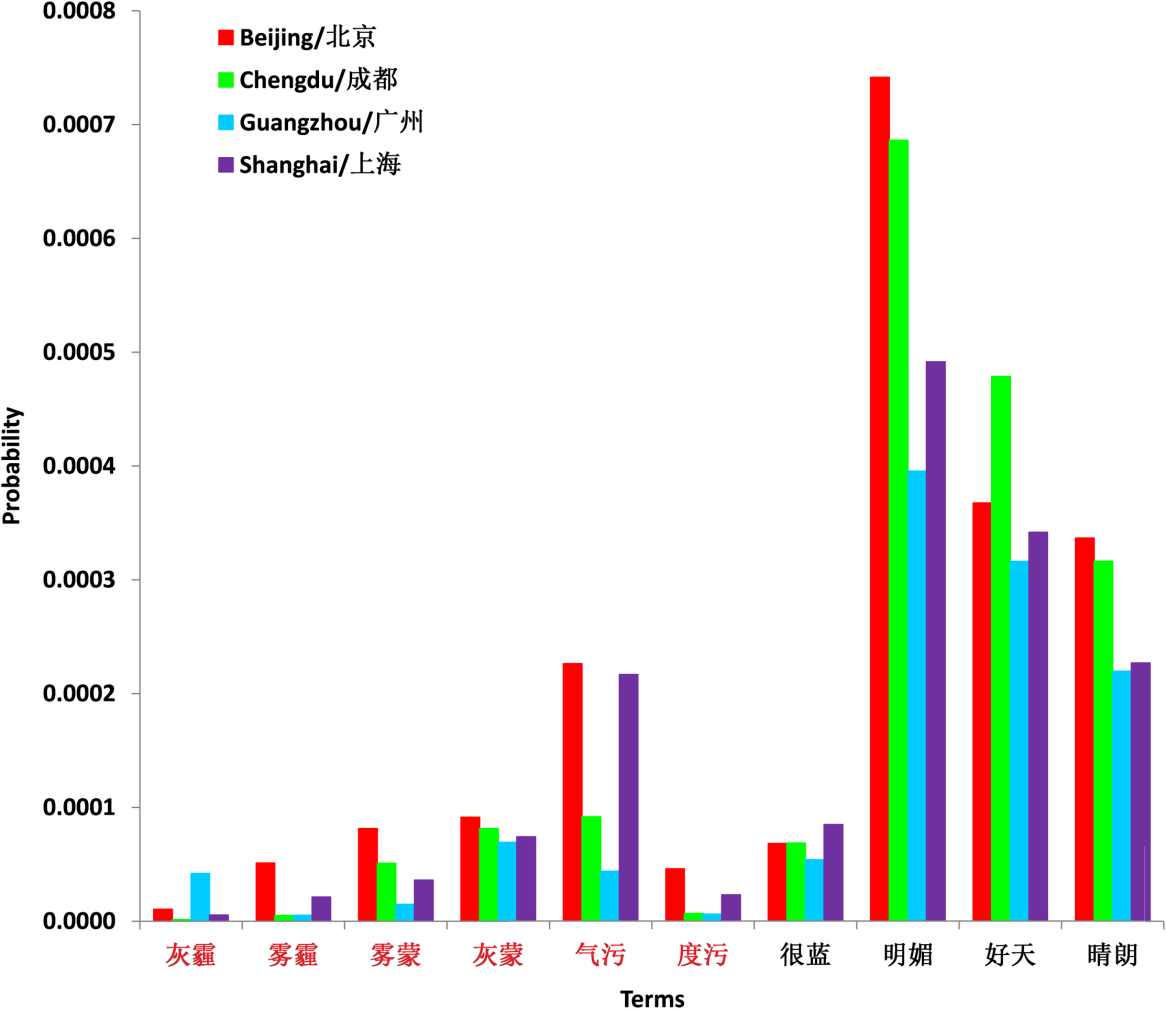
Fraction of Weibo posts containing terms strongly correlated with PM_2.5_. From left to right, positively correlated terms: “dust-haze,” “haze,” “misty,” “dusky,” “air pollution,” “degree of pollution”; and negatively correlated terms: “very blue,” “bright,” “good day,” and “sunny.”

For Beijing, we manually examined 500 terms with the highest scores, including both positively and negatively correlated terms (Fig 1; also see S1 Table for the complete list of the top 500 bigrams from training data in Beijing). We subjectively judged about 95% of them to be air quality or weather-related terms. Some obvious positively correlated examples include “haze”, “fog”, “dusky”, and “cannot breathe”; negatively correlated examples include “sunny”, “raining”, and “blue sky”.

High scoring terms in the other three cities were less obviously related to air quality. For Shanghai, the top 20 terms were related to air quality or weather, all showing positive correlations with PM_2.5_. However, many high scoring terms beyond the top 20 had no apparent relationship to air quality or weather. Selected terms in Guangzhou and Chengdu had even less apparent connection to air quality. The less obvious correlation of high scoring terms in Shanghai and Guangzhou probably related to the less severe air pollution level in the two cites comparing with Beijing. The average daily PM_2.5_ concentrations in Shanghai and Guangzhou during the evaluation period is nearly half of the corresponding value in Beijing (53.4 μg/*m*^3^ in Shanghai and 54.5 μg/*m*^3^ in Guangzhou versus 100.1μg/*m*^3^ in Beijing, see Table 1). More importantly, the frequency of extreme polluted day with the measured daily mean PM_2.5_ concentration greater than 300 μg/*m*^3^ in Beijing is much higher during our evaluation period than the other two cities (in total 19 days in Beijing versus 3 days in Shanghai and only 1 day in Guangzhou), which intuitively has the higher chance to trigger people to directly complain the bad air quality at social media platform. The possible reason for the less direct air quality related high scoring term in Chengdu is due the much smaller sample size comparing with other cites (with average daily Weibo posts around 21000 versus 89000 in Beijing, 83000 in Shanghai and 81000 in Guangzhou, see Table 1).

Some of the terms with high scores merit attention. For example the term “U.S. Embassy” in Beijing and “U.S. Consulate” in Shanghai and Chengdu rank within the top 100 terms in their respective cities, with positive correlations to PM_2.5_. This may indicate an influence from the air quality report from the U.S. embassy or consulate. The reasons for positive correlations in Beijing between PM_2.5_ and terms such as “highway closure” and “download this app” are less readily apparent. For example, it is unclear whether bad visibility caused by pollution was associated with any “highway closures,” and whether pollution made users more likely to download apps showing the AQI.

Another observation is that season-related terms show up frequently in Guangzhou’s list of terms with the highest scores, but are less pronounced in the other cities. Terms related to winter, including winter clothes, tend to have positive correlations with the PM_2.5_. The seasonal nature of word choice in Guangzhou makes it less clear whether word choice such as “鼻塞”(stuffiness) or “喉咙痛” (sore throats) is prompted by higher PM_2.5_ itself, or by seasonal illnesses that tend to peak in the winter.

### Estimation of PM_2.5_ from Weibo Data

We apply the incremental approach (Algorithms 1 and 2) to the learning dataset for each city to test which terms should be included in the final ADI used to infer the PM_2.5_ values. This approach selected a different number of terms for the FTS for each city, as shown in Table 2. Then we apply the linear model described in Eq 5 with the learned parameters to the reserved test data to see how close the fit is to the observed PM_2.5_ values. Correlations between the ADI-derived PM_2.5_ and observed PM_2.5_ in each city are shown in Table 2.

**Table 2:** Performance of Air Discussion Index in estimating observed PM_2.5_.

City	Number of terms in FTS^a^	Learning Period (valid days)	R^b^: learning period	Validation Period (valid days)	R^b^: validation period
Beijing	20	Jul 23, 2011 –Dec 31, 2012 (438)	0.805	Jan 1, 2013 –May 15, 2013 (438)	0.882
Shanghai	12	May 12, 2012 –Dec 31, 2012 (189)	0.737	Jan 1, 2013 –May 15, 2013 (51)	0.633
Guangzhou	42	Jul 26, 2011 –Jan 31, 2013 (349)	0.649	Feb 1, 2013 –May 15, 2013 (113)	0.425
Chengdu	18	Jul 2, 2012 –Jan 28, 2013 (136)	0.853	Feb 1, 2013 –May 15, 2013 (51)	0.361

^a^FTS: Final Term Set

^b^R: Correlation Coefficient

**Fig 2:**
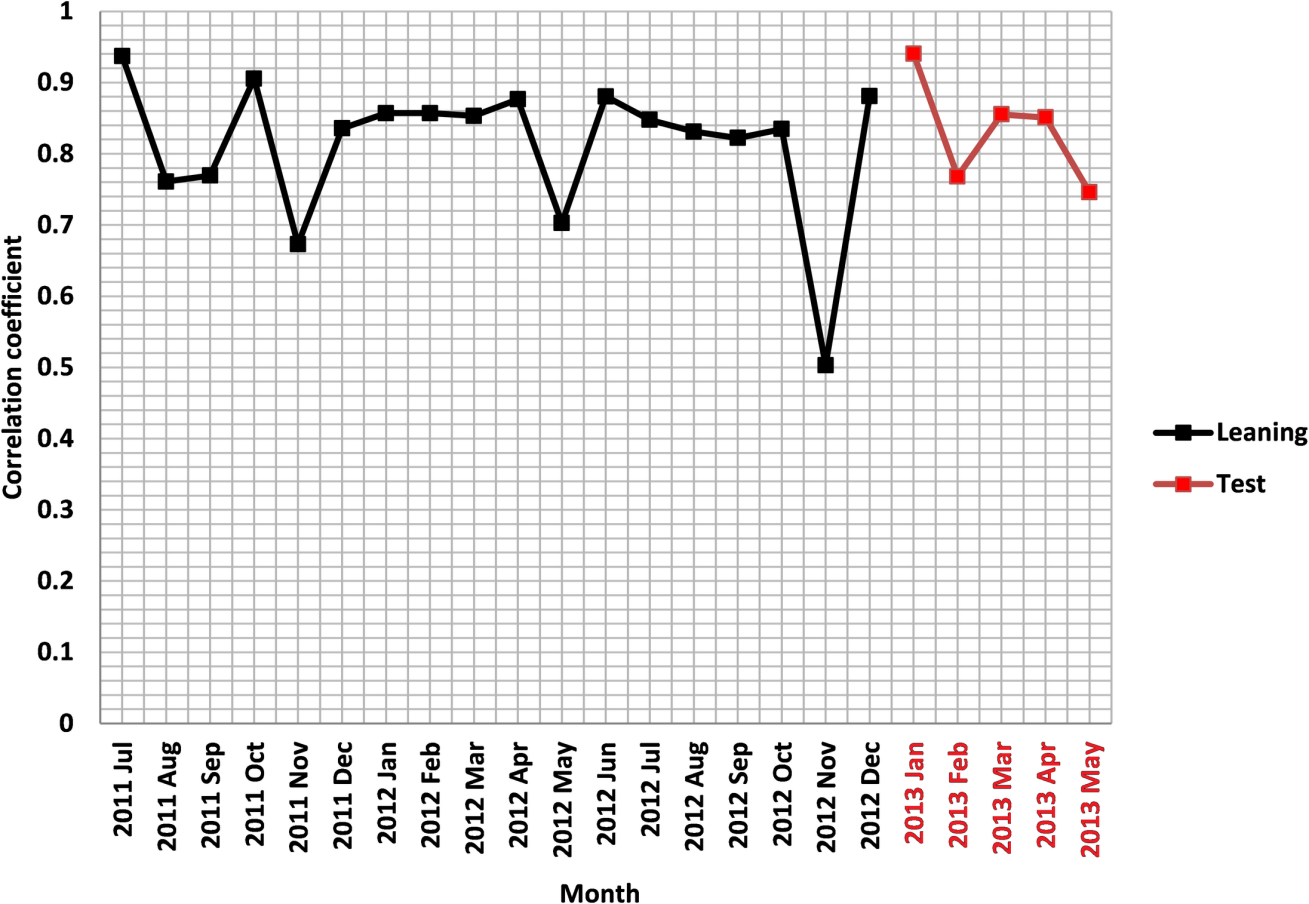
Correlation between observed and estimated PM_2.5_ for Beijing during learning (black) and testing (red) period.

**Fig 3:**
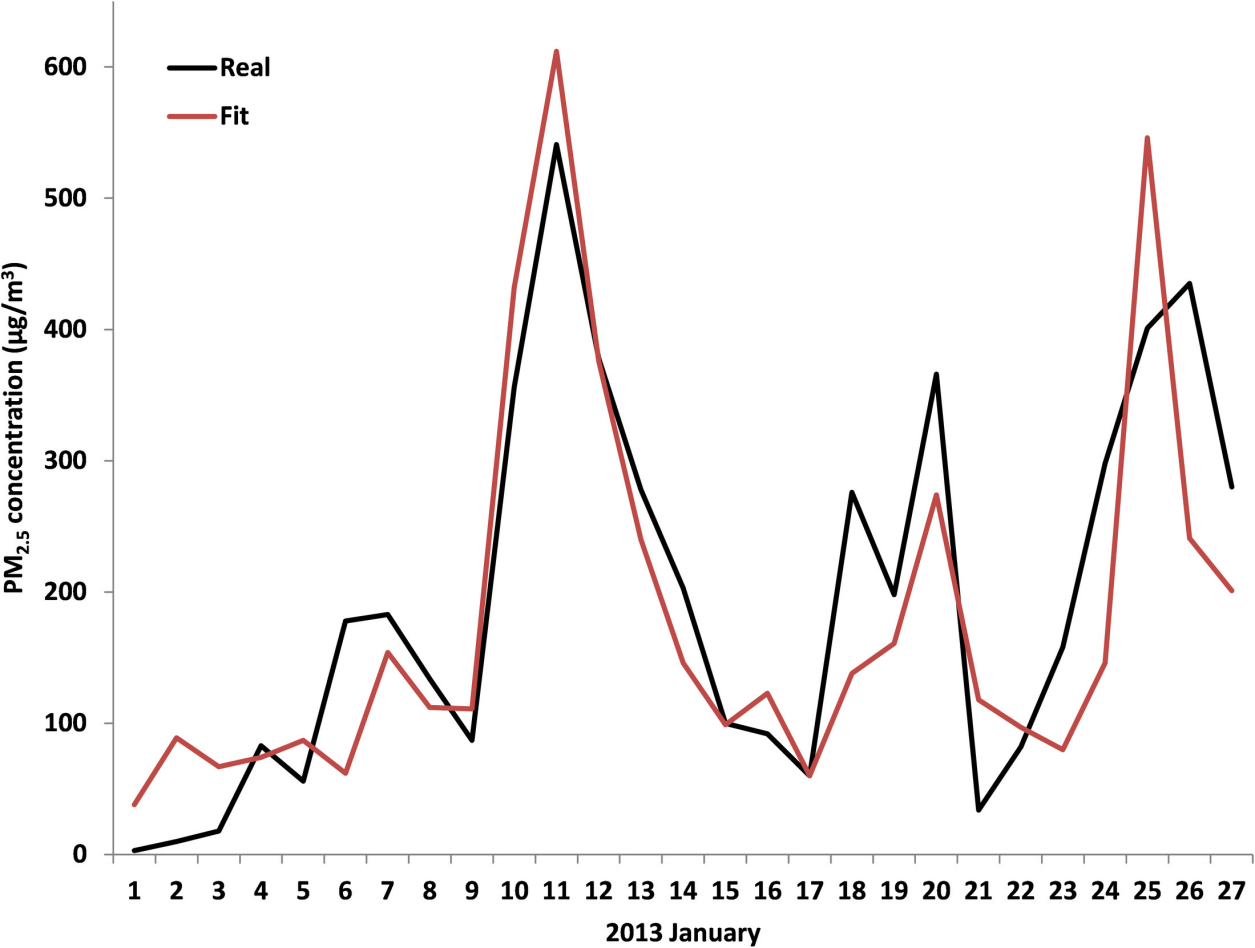
Comparison of ADI model estimates against U.S. Embassy reported PM_2.5_ concentration for Beijing.

Among the four cities, the infer ADI from Weibo posts at capital city Beijing achieved the strongest performance in estimating observed PM_2.5_. In Beijing, there were 438 days of learning data points and 90 days of reserved test data set, and the algorithm selected the 20 terms most correlated with PM_2.5_ at the U.S. Embassy for the FTS of the ADI (see S1 File for the complete list). The correlation between the ADI- derived and the observed PM_2.5_ value for the learning data set is 0.81 and for the validation data set the correlation is 0.88 (Table 2 and Fig 2). The correlation was especially strong in January 2013 (Fig 3), a month with particularly high levels of pollution, suggesting the ADI may become especially useful as air quality worsens. Since January is included in the validation period, it results in a higher correlation for the validation period than the learning period. Applying the same approach to construct an ADI for the other three cities yields less accurate fits of their PM_2.5_ than what was achieved in Beijing. The correlation between the fit and the observation for validation periods were 0.63, 0.43, and 0.36 for Shanghai, Guangzhou, and Chengdu, respectively (Table 2).

**Fig 4:**
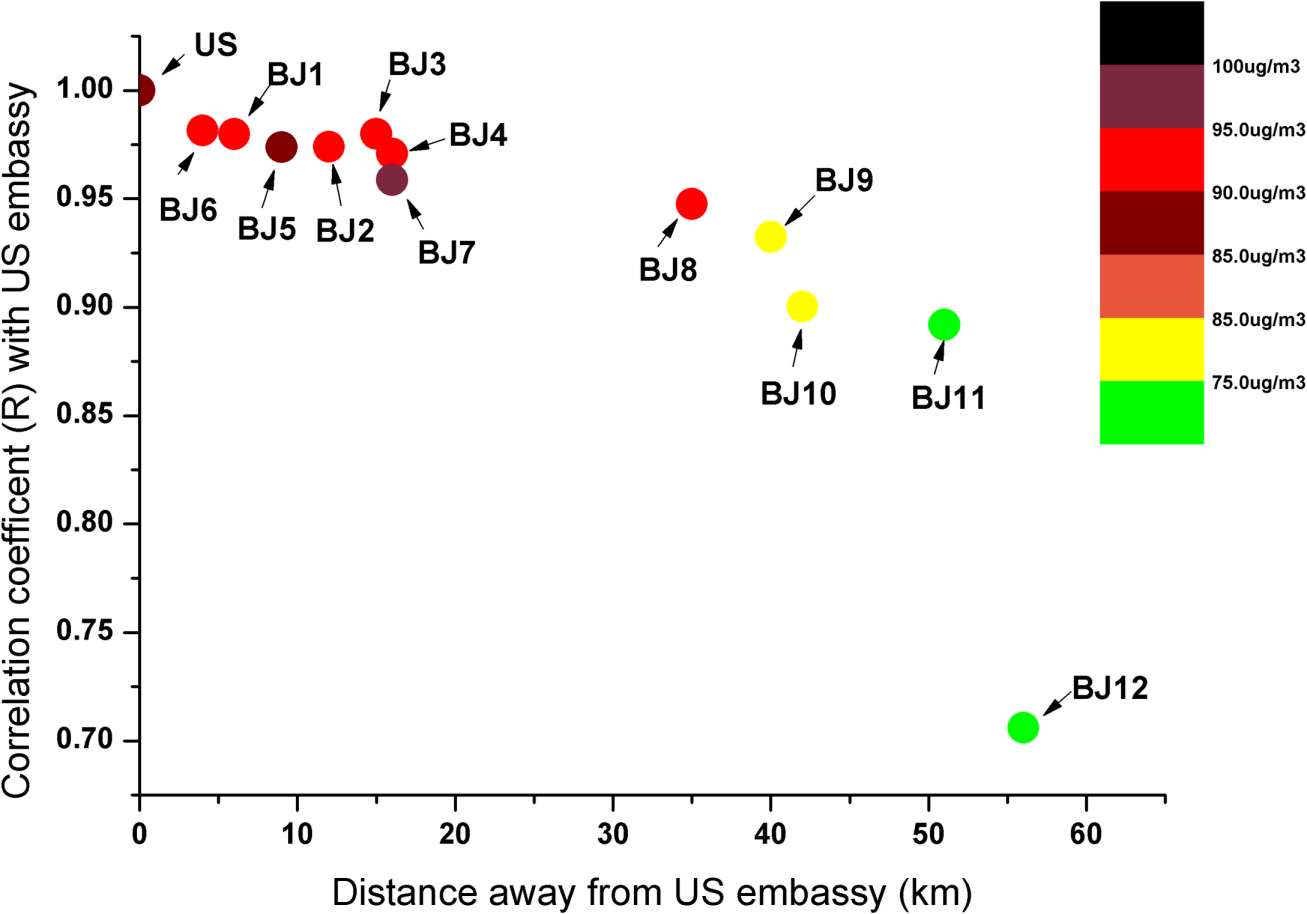
Correlation of daily PM_2.5_ concentrations between U.S. Embassy and 12 BJ-BEP sites. The location of the sites is provided in S1 Fig. The color bar is the annual mean concentration.

The performance achieved by the ADI in estimating Beijing’s PM_2.5_ concentration is especially impressive given fact that the ADI was constructed based on observations at a single monitor (i.e. hourly twitter data from U.S. Embassy), which may not represent the air quality experienced by all Weibo users inside the geographic boundary of Beijing. A correlation analysis between PM_2.5_ at the U.S. Embassy (North East of downtown; see S1 Fig) and the twelve Beijing Environmental Protection Bureau (BJ-EPB) maintained monitoring sites was given in Fig 4 to better understand the extent to which the embassy data represent air quality conditions throughout Beijing area during different seasons (see S2 Fig for the measured PM_2.5_ concentrations at different sites). For 2013, the correlations between daily mean PM_2.5_ at the U.S. Embassy and the other urban sites, especially the eight sites located inside the 6^th^ Ring Road (BJ1-BJ8, which is considered as the core area in Beijing and has more than 75% of total residents) are very high (0.95–0.98) and with quite similar pollution levels (90–100 μg/*m*^3^). Even for the background site BJ12, which is located at the North West upwind direction and around 60 km away from U.S. embassy, the correlation is not low (R = 0.70) and the annual mean PM concentration is also relatively high (69μg/*m*^3^) Since the air quality in Beijing is dominated by regional sources during episode days [36], the spatial different is not that obvious at those days so that people intend to infer the same bad air at Weibo at different geographic locations in Beijing.

**Fig 5:**
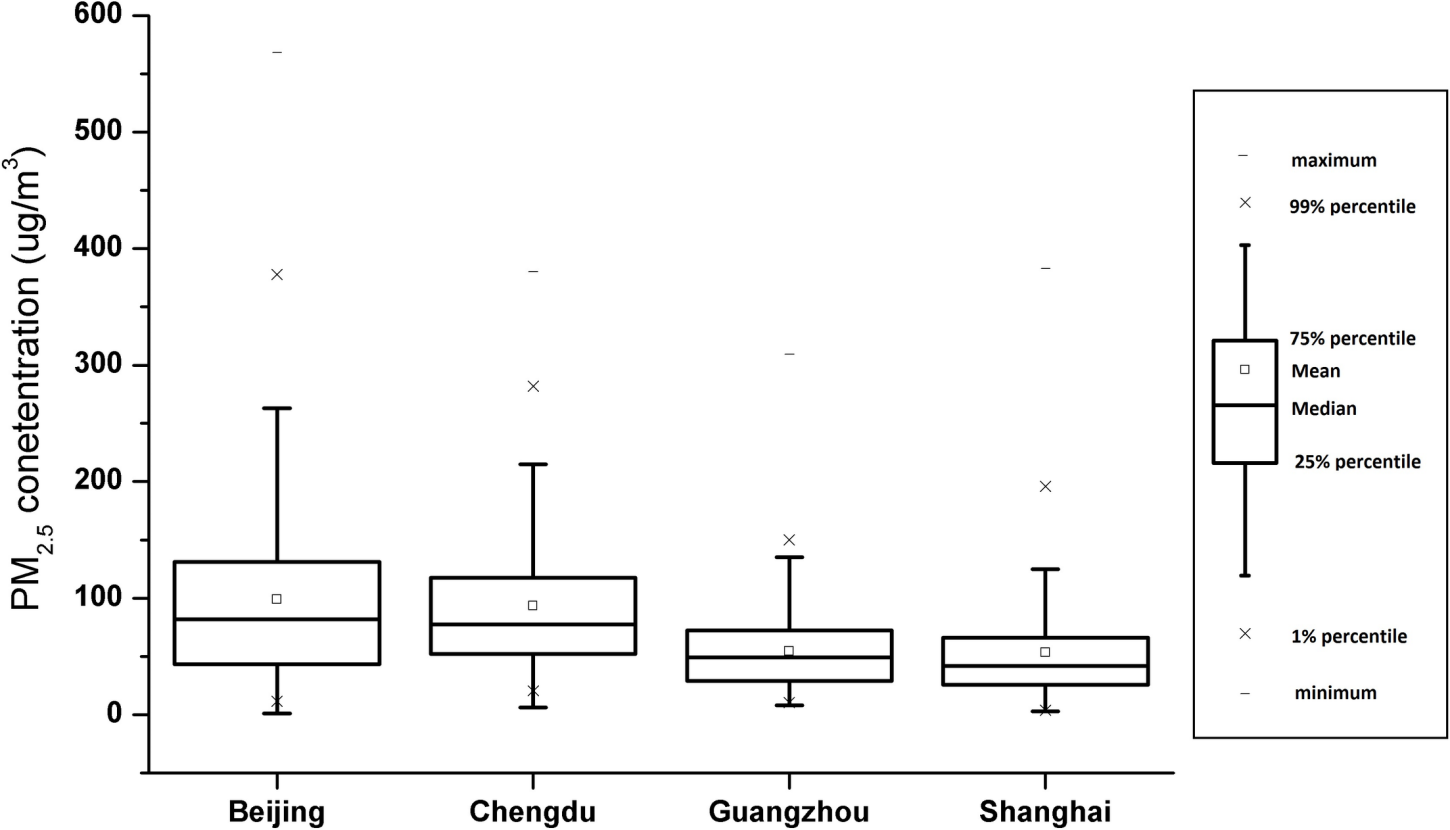
Box plot of daily PM2.5 concentration (μg/*m*^3^) reported in different U.S. Embassy (Consulate) in China.

The Beijing ADI also differs from other cities in having both negatively and positively correlated terms ranked among the most influential terms comprising the index. Three reasons might explain the distinctions between Beijing and the other cities. First, Beijing has the highest and most variable PM_2.5_ levels among the four cities (Fig 5). The larger the variance of the response variable, the easier it is to get rid of the noise terms which coincidentally has similar variance as the response variable [29]. Also, the large variance from a high mean may make it more newsworthy for Weibo users to comment on days with relatively clean air quality in Beijing. Secondly, users in Beijing are more interested in the air pollution issue than other cities (Fig 1). As a result, the key terms have obvious fiuctuations related to air quality. This property fits the motivation of our linear regression method. Finally, we have a longer data record, or learning period, for Beijing than for the other three cities (Table 2).

Besides PM_2.5_, we also attempted to apply this method to PM_10_ (particles smaller than 10 microns in diameter) data in Beijing. However, the resulting ADI achieved a correlation of only 0.56 for PM_10_. PM_2.5_ particles are far more potent than larger particles (which together comprise PM_10_) in attenuating visible sunlight (thereby forming a visible haze and reducing visibility) and impacting human health, and PM_2.5_ is the main basis for air quality alerts. These factors may contribute to PM_2.5_ being more strongly associated than PM_10_ with the content of Weibo posts.

### Comparison with Similar Studies

Our work builds on a corpus of research analyzing both Weibo data and air quality data to measure social and environmental phenomena. We used the pointillism approach for natural language processing of social media to track trends and discover memes. This method, unlike the conventional approach starting from the pre-selected terms, discovers the key terms related to air quality based on mean correlations. In one similar study to utilize Weibo data and device web data to predict pollution related health hazards in China [37], the researchers chose a subset of 38 predefined terms, which have high correlation with API, and used them to monitor the health situation of Weibo users. In another recent Weibo data mining study, the researchers collected social media messages about “outdoor air pollution” in Beijing in 2012 by using the “advanced search” tool in Sina API based on keywords “Beijing” and “air pollution” to do geo-targeted spatiotemporal analysis and infer the AQI in Beijing [38]. Predefined terms are biased by researchers’ opinions and are less successful at discovering the new syndromes or capturing new events. On the other hand, the terms we use here are collected objectively from 40 million terms (bigrams) in our database. Those terms can be updated periodically.

The choice of Chinese words segmentation matters for Weibo posts analysis. Whether to use monograms, bigrams, trigrams, quadgrams and so forth for key term extraction is a trade-off between efficiency and accuracy. Multi-grams extraction tends to contain more information in each search but requires more computation resources, while the monograms search is efficient but cannot give us order information of the phrase. Unlike the similar studies [28–29] to use trigrams to extract topics from Weibo posts, we used bigrams in this study due to the more manageable computational intensity. Furthermore, nearly the same strong correlations were achieved with PM_2.5_ data by using bigram extraction instead of longer grams.

Several factors lead this study to achieve somewhat lower correlations between a Weibo-based ADI and PM_2.5_ than the Google flu trends project [1–2] achieved between search queries and flu incidence. First, the Google queries are typically longer and more specific than a bigram. Secondly, the flu trends research used the query’s IP address while we use the user’s registered city to determine location. It was estimated that about 15% of the time, the city claimed by Weibo users differs from the actual location of sending out posts [37–38]. Thus, our data tends to be more noisy in terms of the mislabeling the geographical information of posts. Lastly, our study used only one monitoring site’s data per city, which did not fully represent conditions throughout the city. Even with these data limitations, our approach achieved a mean correlation of 0.81 (learn data) and 0.88 (validation data) for Beijing, compared to 0.90 and 0.97 respectively for the Google flu trends study.

## Conclusion

In this paper, we show how microblogs posted on China’s popular social media “Weibo” can be used as pollution metric to characterizing the local air quality conditions. We considered PM_2.5_ measurements from four Chinese megacities (Beijing, Shanghai, Guangzhou, and Chengdu) together with 112 million posts on Weibo from days in 2011–2013, which can be break into 40 million bigrams to identify the key terms whose frequency was most correlated with PM_2.5_ levels. These correlations are used to construct an “Air Discussion Index” (ADI) for inferring the daily PM levels based on the content of Weibo posts. In Beijing, the capital city of China with the most frequent and long term ambient PM_2.5_ records from U.S. Embassy monitoring station and most abundant Weibo posts related with air quality, we found a strong correlation (R = 0.88) between the ADI and measured PM. In other Chinese cities with lower pollution levels and fewer related Weibo posts, the correlation was weaker. Nonetheless, our results show that social media may be a useful proxy measurement for pollution, particularly when traditional measurement stations are unavailable, censored or misreported.

Our approach derives the terms whose use correlates most directly with pollution metrics, rather than a priori selection of terms. Building upon prior data mining of Weibo messages, we show how meaningful inferences about pollutant conditions can be extracted from noisy social network data, despite the difficulty of computer processing for Chinese text.

To assure accountability in China’s growing pollution problem, additional public information on environmental issues is urgently needed [39]. The ADI marks a foundation for a future of publicly generated air quality metrics drawn from social media data. The methodology presented in this paper offers an important foundation for similar work in other densely populated areas like South and South East Asia with large social media user bases and limitations to air quality monitoring infrastructure.

Currently we extract ADI per city. As future work, we want to extract common ADI that have common correlation among all Chinese speaking cities in pollution related topics. This can help us estimate the air quality even though we don’t have enough posts corpus.

## Supporting Information

S1 File The final term set for each city. (PDF)

S1 Fig Locations of the US Embassy and the Beijing Environmental Bureau monitoring sites in Beijing, China. (PDF)

S2 Fig Box plot of measured PM_2.5_ daily average concentrations (μg/*m*^3^) from U.S. Embassy site and 12 BJ-EPB sites in Beijing in 2013. (PDF)

S1 Table Top 500 bigrams from training data in Beijing. Shown as the correlation coefficient R value and its corresponding bigram.
(PDF)
