# Supplementary material for: Inferring Atmospheric Particulate Matter Concentrations from Chinese Social Media Data
Source: PLoS One. 2016 Sep 20;11(9):e0161389. doi: 10.1371/journal.pone.0161389 (PMC5029919; doi:10.1371/journal.pone.0161389)
Supplement: S1 File — (PDF) [file pone.0161389.s001.pdf]

S1 File. The final term set for each city.

The lists below show the Chinese bigrams chosen for the final term set for constructing the Air Discussion Index in each city. Since each Chinese bigram does not necessarily represent a word or phrase, it is not always possible to translate a bigram directly into an English meaning. We list the bigrams here with associated with corresponding English translation only with clear meaning.

Beijing (20 terms)

雾蒙(foggy/misty) 蒙的(muddy) 度污(degree of turbidity) 毒害(hazard) 阳光(sunshine) 很蓝(very blue) 戴口(wear mask) 轻雾(light fog) 空好 阳天(sunny) 真闷(stuffy) 风嗖 (windy) 微颗 (micro particulate) 迎着 天阳 (sunny sky) 天蓝 (blue sky) 好蓝 (very blue) 气晴 京雾 无云 (cloudless)

Shanghai (12 terms)

不健 (not health) 极不 度污 (degree of turbidity) 污染 (pollution) 雾蒙 (foggy/misty)雾霾 (foggy/haze) 灰蒙 (grey/haze) 空气(air) 口罩(mask) 戴口 有雾 (foggy) 有毒 (hazard)

Guangzhou (18 terms)

灰霾 (grey/haze) 吹着 (blow) 凉快 (cool) 的天 费空 雨后 (after rain) 运指 吹吹 榜为 凉席 存储 吹风 (windy) 州劳 有雾 (foggy) 天空 (sky) 处苍 签到 下云

Chengdu (42 terms)

的天 (-like day) 天空(sky)鼻酸 (sour nose) 雨过 (passing the rain) 都旅 谴责 (blame) 和誓 睡在 受这 天充 却那 使一 小爬 厅在 天夜 将廖 动律 乱世 鼻塞 (stuffiness) 蓝天 (blue sky) 个天 击终 师网 出门 (outdoor) 盘签 凉爽 (cool) 越多 网旗 很凉 (cool weather) 純妹 大雾 (heavy fog) 雾天 (foggy) 大太 你就 霾天(haze) 短片 将你 风云 完雨 下暴 倾盆 (pouring) 皮草
