# Supplementary material for: Inferring Atmospheric Particulate Matter Concentrations from Chinese Social Media Data
Source: PLoS One. 2016 Sep 20;11(9):e0161389. doi: 10.1371/journal.pone.0161389 (PMC5029919; doi:10.1371/journal.pone.0161389)
Supplement: S1 Fig — (PDF) [file pone.0161389.s002.pdf]

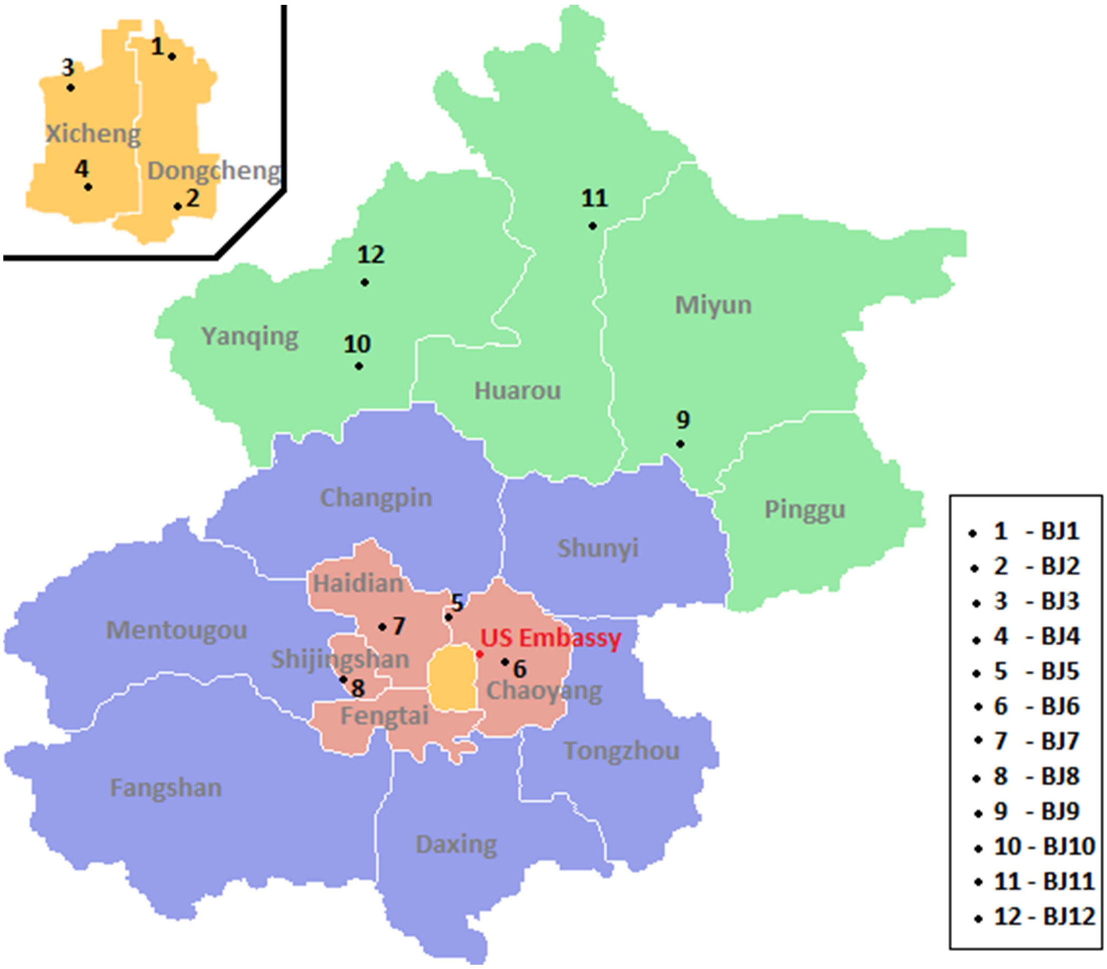

S1 Fig. Locations of the US Embassy and the Beijing Environmental Bureau monitoring sites in Beijing, China*

(*The background map is the adaption of administrative division of Beijing https://commons.wikimedia.org/wiki/File:ColorBeijingMap.png with the yellow area as the inner city inside the 2nd Ring Road, the pink area as the urban area between 2nd and 5th Ring Road, the purple area as inner suburbs linked by the 6th Ring Road and the green area as outer suburbs within city limits. The grey words are the names of the administrative division of Beijing.)
