# Supplementary material for: Inferring Atmospheric Particulate Matter Concentrations from Chinese Social Media Data
Source: PLoS One. 2016 Sep 20;11(9):e0161389. doi: 10.1371/journal.pone.0161389 (PMC5029919; doi:10.1371/journal.pone.0161389)
Supplement: S2 Fig — (PDF) [file pone.0161389.s003.pdf]

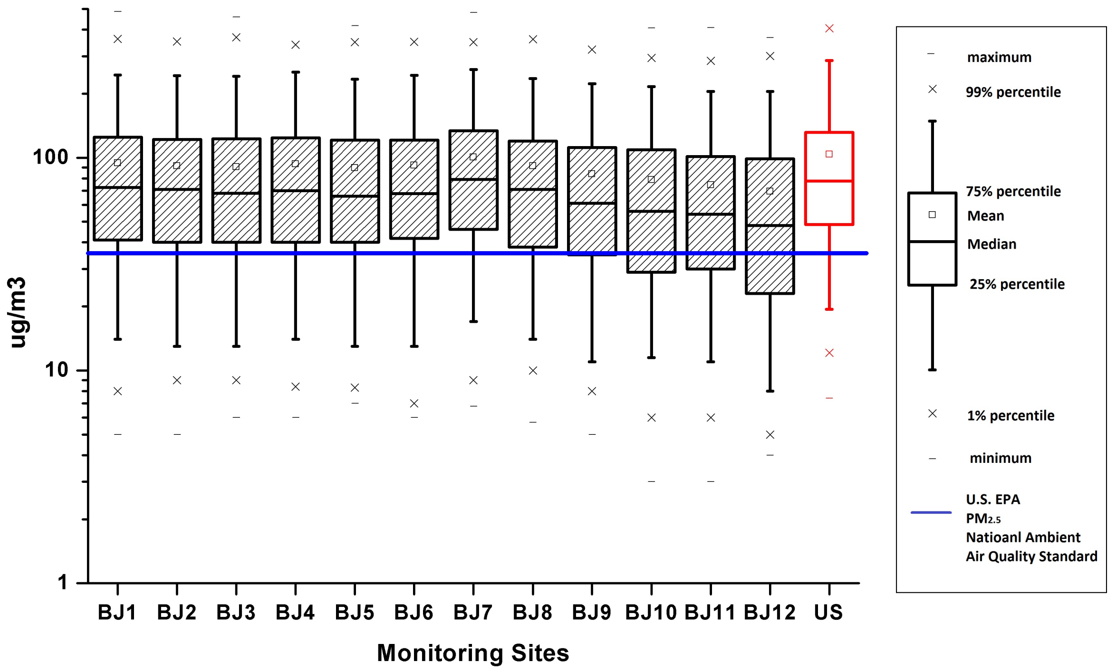

S2 Fig. Box plot of measured $PM_{2.5}$ daily average concentrations (μg/m^3) from U.S. Embassy site and 12 BJ-EPB sites in Beijing in 2013*.

(*Refer the legend for box plot to show the statistic distribution of data, where the symbol □ represents the mean, the three ▬ in the box represent the 25% percentile, median, and 75% percentile respectively, the two × represents the 1% percentile and 99% percentile respectively, the two − represents the maximum and minimum respectively. The blue line is the US standard for $PM_{2.5}$ annual mean.
