# Supplementary material for: Inferring Atmospheric Particulate Matter Concentrations from Chinese Social Media Data
Source: PLoS One. 2016 Sep 20;11(9):e0161389. doi: 10.1371/journal.pone.0161389 (PMC5029919; doi:10.1371/journal.pone.0161389)
Supplement: S1 Table — Shown as the correlation coefficient R value and its corresponding bigram. (PDF) [file pone.0161389.s004.pdf]

S1 Table. Top 500 bigrams from training data in Beijing. Shown as the correlation coefficient R value and its corresponding bigram.

1 - 100	101- 200	201 - 300	301 - 400	401 - 500
0.608 雾蒙	0.348 迷雾	0.275 光透	0.239 自美	0.217 多星
0.597 蒙的	0.346 刺眼	0.274 中等	0.239 光太	0.217 沉的
0.566 度污	0.346 雾封	0.273 大霧	0.237 天跳	0.217 有蓝
0.562 毒害	0.345 气弥	0.273 个艳	0.237 分剜	0.217 很干
0.550 阳光	0.344 气污	0.273 个阳	0.237 去晒	0.217 霞很
0.544 很蓝	0.340 这雾	0.272 的朝	0.237 点秋	0.217 空特
0.529 戴口	0.340 破天	0.272 阴天	0.237 光暖	0.216 晨大
0.527 轻雾	0.337 雾大	0.272 太闷	0.237 京阳	0.216 云很
0.520 空好	0.337 朗的	0.271 阳出	0.236 这闷	0.216 宾外
0.513 阳天	0.336 晨雾	0.271 阴有	0.236 真冷	0.216 风还
0.504 真闷	0.336 晒晒	0.271 秋高	0.235 阴阴	0.216 玛冷
0.490 风嗖	0.335 空万	0.271 阴霾	0.235 高气	0.215 常起
0.472 微颗	0.334 雾黄	0.270 京难	0.235 罗店	0.215 风一
0.470 迎着	0.333 照在	0.270 是阴	0.235 沉沉	0.215 航班
0.462 天阳	0.332 万里	0.270 气缭	0.234 冷风	0.214 还悠
0.462 天蓝	0.332 很晴	0.269 雾下	0.234 好美	0.214 检表
0.462 好蓝	0.331 空湛	0.269 云好	0.234 剜掉	0.214 憋闷
0.456 气晴	0.331 阳高	0.267 光照	0.234 心行	0.214 滴都
0.452 京雾	0.330 毒气	0.267 闷的	0.234 风中	0.214 光特
0.445 无云	0.329 媚啊	0.266 空灰	0.234 光普	0.214 天朗
0.443 的阳	0.329 大晴	0.265 湛蓝	0.233 阴沉	0.214 烂的
0.443 光真	0.328 雾真	0.264 里无	0.233 值卡	0.213 同妳
0.442 有雾	0.328 雾有	0.263 这阳	0.232 气让	0.213 空气
0.435 光很	0.328 速封	0.262 天闷	0.232 外晴	0.213 气灰
0.433 雾了	0.327 能见	0.260 天天	0.232 斗七	0.213 浊的
0.429 个雾	0.327 浓雾	0.260 好闷	0.232 见蓝	0.212 晨光

0.427 雾很	0.327 么蓝	0.260 糟糕	0.232 闷闷	0.212 刮着
0.427 真蓝	0.326 气糟	0.260 风吹	0.232 点闷	0.212 线封
0.426 好晴	0.324 白云	0.258 天空	0.231 受阳	0.212 候吓
0.426 天雾	0.324 静岭	0.258 着阳	0.230 晒被	0.212 着毒
0.425 雾朦	0.323 朦的	0.258 来场	0.230 的蓝	0.212 不下
0.424 光明	0.323 在雾	0.257 晚霞	0.230 着夕	0.211 气赶
0.423 媚的	0.323 见度	0.257 成雾	0.230 求雨	0.211 拂面
0.421 起雾	0.321 气好	0.257 西下	0.230 雾也	0.211 气里
0.421 下雾	0.320 雾中	0.256 闷死	0.229 样闷	0.211 开灯
0.418 有毒	0.319 阳好	0.256 给室	0.229 都看	0.210 天的
0.417 重污	0.318 毒面	0.256 湿度	0.228 来气	0.210 光甚
0.416 雾的	0.318 云淡	0.256 风挺	0.228 通安	0.210 言小
0.415 雾气	0.318 必佩	0.255 物浓	0.228 儿冷	0.210 遍啦
0.415 雾都	0.317 雾里	0.255 阳暖	0.228 晨的	0.210 小雾
0.409 霾的	0.317 朦朦	0.254 晃眼	0.228 雾是	0.210 都冻
0.408 光好	0.316 雾不	0.254 云朵	0.228 么晴	0.210 格外
0.407 议佩	0.313 弥漫	0.253 光下	0.227 碧空	0.210 钟奖
0.404 极不	0.312 气笼	0.253 阳中	0.227 这破	0.210 风有
0.403 光灿	0.312 耀眼	0.253 污浊	0.226 天边	0.210 天很
0.401 雾笼	0.311 笼罩	0.252 霾中	0.226 桑拿	0.210 凡到
0.400 雾霾	0.311 陽光	0.252 鬼天	0.226 刘鸿	0.209 容尽
0.399 上雾	0.311 明媚	0.252 洒在	0.226 阳当	0.209 报明
0.395 面雾	0.310 的晴	0.252 这阴	0.226 合郊	0.209 真漂
0.393 灰蒙	0.310 雾太	0.252 蓝湛	0.226 真脏	0.209 风天
0.392 风和	0.310 好天	0.251 大闷	0.226 灰沉	0.209 彩真
0.391 雾霭	0.310 处好	0.251 挺风	0.226 罩了	0.209 载此
0.390 雾锁	0.308 难得	0.251 灰朦	0.225 于下	0.209 吹乱
0.389 这空	0.308 闷热	0.251 这鬼	0.225 么闷	0.209 您手
0.388 日丽	0.305 的云	0.251 冻感	0.225 黄奕	0.209 命烈
0.387 晴好	0.305 阳无	0.251 品宣	0.225 晒的	0.209 天阴
